# Psychometric Properties of the Psychological State Test for Athletes (TEP)

**DOI:** 10.3389/fpsyg.2020.566828

**Published:** 2020-10-15

**Authors:** Patricia Díaz-Tendero, M. Carmen Pérez-Llantada, Andrés López de la Llave

**Affiliations:** ^1^Health Psychology, National Distance Education University (UNED), Madrid, Spain; ^2^Department of Behavioral Sciences and Methodology, Faculty of Psychology, National Distance Education University (UNED), Madrid, Spain

**Keywords:** anxiety, emotional state, motivation, performance, pre-competition, psychological evaluation

## Abstract

This study has two objectives: to validate an adapted online version of the Psychological State Test (TEP, in its Spanish acronym); and to assess differences in pre-competitive psychological state profiles between amateur and professional athletes in team sports. The TEP questionnaire is an instrument which is used to assess, in a quick and simple fashion, the psychological state of athletes prior to competing. Its psychometric properties were evaluated by means of an analysis of internal consistency, an Exploratory Factor Analysis and a Confirmatory Factor Analysis. The EFA’s results showed a factorial structure consisting of two principal factors and reliability coefficients, both globally and at the factor level, which can be considered acceptable (global α = 0.81; Factor 1 α = 0.85; and Factor 2 α = 0.73). The CFA confirmed the model proposed by the EFA so that the items were distributed around these factors, giving rise to one factor which includes variables that have a positive relationship with performance, and another with variables that negatively affect performance. Meanwhile, regarding the difference between the pre-competitive psychological state of amateurs and professionals, professional athletes presented higher levels of Motivation (p = 0.5 and d = −0.23). It is concluded that the TEP is a suitable tool for the evaluation of pre-competitive psychological states. However, in future research, this study should be complemented by analyzing the TEP’s predictive validity in terms of the performance of athletes and/or teams, as well as its use as a tool available to athletes and coaches.

## Introduction

Various studies have shown how the emotional states presented by athletes before competing are determining factors in regard to their performance (Cerin, 2003; Hanin, 1997, 2000a,b). According to Lazarus (2000), the function of emotions is to facilitate adaptation to different environmental conditions and, by extension, when referring to athletes, to facilitate their performance. Moreover, this author considers that the positive or negative influence of emotions on performance will depend on the threat-challenge balance which the athlete perceives in the situation they are about to face, as well as the resources they possess in order to handle this situation. From a different perspective, but in agreement with the importance of the athletes’ personal assessment, Hanin (1997) presented his model: “Individual Zone of Optimal Functioning” (IZOF), in which he proposes that emotional states prior to competition can affect athletes in different ways. Thus, each athlete would have an Individual Zone of Optimal Functioning (IZOF), defined by a set of emotions which may vary in terms of their intensity (high, moderate and low), and which are functional or not depending on the athlete.

Hanin (2000a) also proposed the IZOF-Emotion model, based on which he developed the “Emotional State Profile” (ESP-40) in which he identifies four emotional categories based on a list of 40 adjectives which helped to define the pre-competitive emotional state of athletes. These four categories of emotional states are: positive emotions which improve performance (P+), negative emotions which improve performance (N+), positive emotions which impair performance (P-), and negative emotions which impair performance (N-). Feeling happy and vigorous are considered by Hanin to be emotional states which improve performance (P+), while feeling unhappy and sad are identified as emotions which impair performance (N-).

The psychological state of the athletes as a construct shall be defined by these psychological variables together with the emotional state prior to competition, which has evidenced to have an influence on sports performance. Those psychological variables most studied in the scientific literature for their effect on sports performance are: self-confidence, motivation, stress, arousal levels, anxiety, and mood. To summarize, some data on these variables and their relationship to performance have been included. Self-confidence was used as a synonym for the term self-efficacy. Bandura (1977, 1997) described self-efficacy as the belief that one can master a situation and produce positive results. Campbell and Pritchard (1976) considered motivation as the factor that induces people to make the decision to start an activity, to put forth a certain amount of effort and to persist in it for a certain period of time. In relation to motivation, Clancy et al. (2016) conducted a review on competitive sport motivation between 1995 and 2016. In this study, the large number of studies that tested the importance of the motivation variable in influencing athletes’ performance were taken into account. Concentration, understood as the maintenance of the attentional conditions for the duration of the sporting activity, promotes the athletes’ processing of information (Boutcher, 2008). The arousal level variable refers to the level of physical and psychological activation of athletes while practicing sport (Malmo, 1959). The search for the optimal level of arousal, as well as the effects of high and low arousal level on performance, has been widely studied by many authors (e.g., Weinberg and Gould, 2007; Yerkes and Dodson, 1908). Stress refers to the perception of pressure by athletes when confronted with the demands of a situation, and they must adapt their responses under conditions in which failure can bring about serious consequences (McGrath, 1970). Finally, there is the anxiety variable. Nuñez and Garcia-Mas (2017) conducted a systematic review of the relationship of anxiety in sport. Although they concluded that no sufficient empirical and/or experimental evidence existed to explain the relationship between anxiety and sporting performance, the authors clarified that *“as a result of the volume of studies carried out on this subject, at the “popular” level anxiety is a problem that affects performance, given the large number of studies focused on dealing with this problem.”* One reason for the possible cause of these difficulties of finding evidence of the relationship between anxiety and performance is the disparity in theoretical frameworks and the difficulty of systemising the concept of “performance.” In this sense, the Catastrophe Theory (Hardy, 1990) states that when cognitive anxiety is high, the increase in the activation only improves performance to a certain point, and from this point it would produce a dramatic decline in performance (“catastrophe”), rather than a gradual decrease. Therefore, the activation may produce different effects on performance based on the individual’s level of cognitive anxiety. In this sense and based on the Jones (1991) on the Directionality Theory, Ponseti et al. (2016) found in a study of swimmers that competitive anxiety has a blocking and debilitating effect on sporting performance. The authors concluded that the most important component is the cognitive factor, associated in turn with concern about performance. Authors who have studied the psychological state of athletes in the days and hours before a competition have demonstrated that, during the days, hours and even minutes leading up to competition, it is optimal that athletes present the physical and psychological state which enables them to achieve the best possible performance according to their sporting circumstances (e.g., Buceta, 1998; Cerin, 2003; Hanin, 1997, 2000a,b). Martens et al. (1990a) determined that cognitive anxiety and somatic anxiety functioned differently depending on the time interval before the sporting event. They concluded that both types of anxiety had different effects from two days before the competition up to 24 h afterwards. Meanwhile, Buceta et al. (2003) carried out research to determine the psychological state of public marathon runners between 65 and 12 h before the event. To do so, they used the CSAI-2 questionnaire (Martens et al., 1990a) which considers variables such as somatic anxiety, cognitive anxiety and self-confidence. They found that the psychological profile of these marathon runners before the race was defined by medium-low scores for somatic anxiety and cognitive anxiety, and a medium-high score for self-confidence. This suggested that, in general, public marathon runners, in the days l eading up to the event, managed to keep stress related to the race at an acceptable level and perceived that they could achieve their objectives.

Over the past few years, a number of studies have emerged that have investigated the momentary mood states of Brazilian football players in pre-competition situations (e.g., Silva, 2017; Souza, 2014). They found that football players have a common profile in the pre-competition moments determined by interest, happiness and hope. They also confirmed that the mood state prior to competition differed between players depending on their position in the game, so players in defence and forward positions presented different psychological profiles (Bueno and Souza, 2019). The authors drew on The Present Mood States List (PMSL), proposed by Engelmann (1986, 2002) to evaluate mood states resulting from wide empirical research performed in Brazil for these studies.

There are other several tools which have been used to assess the psychological state of athletes before competing. One of the most widely used is the Profile of Mood States (POMS) from McNair et al. (1971). The POMS is a test which consists of a list of multidimensional adjectives. In psychology, tests based on adjective lists are generally used to measure feelings, affects and mood states (Ávila and Giménez de la Peña, 1991). Based on the POMS, Morgan (1980a, b)Morgan and Jhonson (1977, 1978) and (Morgan and Pollock, 1977) identified the “Iceberg Profile,” which refers to the layout of the scores in graphic form (which resembled the shape of an iceberg) by which the variables: Pressure, Depression, Anger, Fatigue, and Confusion are below the population average, and Vigor above it. In addition, the “Iceberg Profile” was regarded as a predictor of athletic performance before competition (e.g., Nagle et al., 1975). Limitations of this instrument have been pointed out, among which we can highlight those by Beedie (2005): (a) its factors have a predominantly negative orientation, (b) some items are associated with constructs not related to mood states, and (c) there are difficulties as regards distinguishing between emotions and mood states when using the POMS with athletes. Schacham (1983) found that individuals under conditions of stress or pain could take between 15 and 20 min to complete this questionnaire, which could be significantly limiting in pre-competitive sports contexts. In recent years, a number of tools based on the POMS have emerged, such as the Interactive Profile of Mood States in Sports (PIED in the Spanish acronym), created by Barrios (2011). This scale includes six lists of adjectives which correspond to each of the POMS scales, and in which athletes must indicate the intensity with which they are perceived on a scale ranging from 0 (“not at all”) to 4 (“very much”). Another such tool is the POMS-VIC, developed by De la Vega et al. (2014). This is an adaptation of the version of the POMS developed by Andrade et al. (2008), in which three scales were used: mood state intensity, mood state valence and mood state control.

As an alternative to the POMS, another tool to assess pre-competitive psychological state which we have previously discussed is the reduced version of the CSAI-2. The CSAI-2R (Andrade et al., 2007) adapted from Martens et al. (1990a), consists of 17 items and has been widely used to assess levels of somatic anxiety, cognitive anxiety and self-confidence in the moments leading up to competition. The main limitation of this instrument is that, compared to other scales or instruments which give a broader profile of the athlete in these pre-competitive moments, the CSAI-2R only provides information on the anxiety (cognitive and somatic) and self-confidence variables.

A different approach which resolves many of the limitations found so far in regard to assessing precompetitive psychological state, is that developed by Buceta (2010). The Psychological State Test (TEP) was created with the objective of assessing the psychological state of athletes in an overall manner. Athletes can complete the test quickly (in under a minute) and at any time. It has mainly been used to better understand a team’s collective disposition (it was initially used with soccer players) and, based on this information, to advise coaches and/or help athletes individually. The TEP is based on the PODIUM questionnaire for marathon runners, which was created in order to help these athletes adapt their psychological state prior to racing (Buceta et al., 2003; Larumbe et al., 2018). The PODIUM questionnaire consists of 20 items grouped into 6 psychological factors or variables: Somatic Anxiety (α = 0.83), Cognitive Anxiety (α = 0.77), Motivation (α = 0.86), Self-confidence (α = 0.72), Physical Perception (α = 0.90), and Social Support (α = 0.74). The response format used was Visual Analog Scales (VAS), from which two opposite adjectives were presented, upon which runners were asked to mark their responses on a 10-centimeter line according to how they felt at the time. Accordingly, the TEP (Buceta, 2010) consists of nine similar visual scales, each of which refers to one item in the questionnaire (9 items in total) and each consisting of two opposing adjectives. Each scale/item refers to a psychological variable related to sports performance (Weariness, General Tiredness, Positive arousal, Motivation, Self-Confidence, Concentration, Negative arousal, Anxiety, and Hostility), and the result is an overall profile of the athlete’s psychological state. Athletes answer by placing an x on the one-hundred-millimeter line which separates both adjectives, depending on how they feel at that moment (self-report). Response coding is obtained by measuring the position of the athlete’s mark on the line, considering each millimeter as a unit starting at 0 and ending at 100, and allowing the athlete’s score on the item to be recognized. The small number of items, as well as the simplicity of the response format, mean that this test is suitable for use in the moments prior to competing, when anxiety levels can limit the athlete’s capacity for self-observation. These limitations in self-observation have been explained mainly by the role of somatic anxiety in these pre-competition moments, with the anxiety felt most intensely in the hours and minutes leading up to the event (Martens et al., 1990a; Buceta et al., 2003).

As previously seen in relation to the different psychological profiles presented by Brazilian football players using the PMSL scale (Bueno and Souza, 2019), one of the possible applications of TEP could be related to the ability to assess whether different groups of athletes (based on gender, age, level of dedication, etc.) present distinct psychological profiles. There are various studies which compare the function of psychological variables related to the performance of different groups of athletes. In terms of comparisons between the influence of psychological variables on amateur (or non-professional) and professional (or elite) athletes, some examples include: Modroño and Guillén’s (2006) study with regard to the motivation variable and the difference between amateurs, professionals, and non-professionals; and Kerr and Pos (1994) study which demonstrated a difference in the psychological mood experience between high level and low level competitive gymnasts, both in the training setting as well as the competition setting.

Although the TEP is a tool which is frequently used by sports psychologists in the applied field, its psychometric characteristics are unknown. The main objective of this paper is to study the psychometric characteristics of the Psychological State Test, through an Exploratory Factor Analysis and a Confirmatory Factor Analysis, in addition to the calculation of its Reliability indices (which will allow us to obtain an approximation as to the instrument’s validity). On the other hand, in order to verify the TEP test, once its factorial structure was confirmed, we chose to propose a secondary objective: the comparison between the pre-competition profiles of amateur athletes and professional athletes in team sports. The aim is to verify whether there are statistically significant differences between the two groups in the moments leading up to competition in relation to the psychological variables assessed by the TEP and, if so, to examine what these differences consist of and whether they are in line with those found in previous studies which have compared psychological variables related to performance among these two groups.

## Materials and Methods

### Instruments

An adaptation of the TEP in an online format was used. A pilot study was carried out with 20 athletes (selected based on the inclusion criteria established). The aim of this was to assess whether the adjectives used to describe the scales of the TEP were understood according to the psychological variable that was intended to be measured, and that words habitually used in the sporting context of the athletes were being used. As a result of this first study, it was found that participants were having trouble differentiating between the following adjectives used in the scales: Positive Arousal, Negative Arousal and Anxiety. One possible cause of this ambiguity regarding the conceptualization of the variables of the initial version of the TEP may be warranted by the author’s description of them. According to Buceta (2010) the variable “positive arousal” emanates from motivation and “negative arousal” from stress. Furthermore, he stated in his text that, with regard to the relationship of these two variables with optimal level of arousal, the following can be taken into account: positive arousal with optimal levels of general arousal and negative arousal with an excessive level of general arousal. We thus consulted with five experts in Sports Psychology (each of whom had more than 10 years of practical experience) in order to assess how the scales which had led to ambiguity could be reconceptualized. This resulted in three changes: the “Positive arousal” scale was reconceptualized as “General arousal,” the “Negative arousal” scale was reconceptualized as “Stress” and the scale initially referred to as “Anxiety” was renamed “Cognitive Anxiety.” The use of a “cognitive anxiety” scale that was more specific than the “anxiety” variable in the initial version of the questionnaire was also supported by the conclusions of Martens et al. (1990a), in which they highlighted that the “cognitive anxiety” variable over “somatic anxiety” indicated levels that were higher and more stable throughout the days and hours prior to competition. Furthermore, a number of the adjectives were modified by means of selecting those which were repeated most among the group of participants in the pilot study and verified by the group of experts. The latter agreed that the “General fatigue” scale should be changed to read “Rest.” It was expected that the positive description of the variable would be met with less resistance from the athletes. Finally, since our interest was in the evaluation of teams, we considered it important to include a scale that referred to the athletes’ perception of team cohesion, given that numerous studies have related high levels of group cohesiveness with a greater perception of collective efficacy within teams (e.g., Heuze et al., 2006; Leo Marcos et al., 2011; Spink, 1990). The results of the pilot study led to the version of the TEP used in this paper, which consisted of 10 items: Rest, Self-Confidence, Motivation, Concentration, Hostility, Mood State, General arousal, Stress, Cognitive Anxiety, and Team cohesion (Table 1).

**TABLE 1:** Comparison between the variables in the original version of the TEP and those in our adaptation.

	**TEP 2010**		**Adaptación TEP 2019**	
**Variables**	**Adjetivos opuestos**	**Variables**	**Adjetivos opuestos**
Cansancio general	Cansado/a – Fresco/a	Descanso	Cansado/a – Con energía
Autoconfianza	Con Confianza – Sin Confianza	Autoconfianza	Con Confianza – Sin Confianza
Motivación	Motivado/a – Desmotivado/a	Motivación	Motivado – Desmotivado
Concentración	Centrado/a – Disperso/a	Concentración	Centrado/a – Disperso/a
Hostilidad	Calmado/a – Enfadado/a	Hostilidad	Calmado/a – Enfadado/a
Desánimo	Contento/a – Triste	Estado de ánimo	Contento/a – Triste
Activación positiva	Activado/a – No activado/a	Activación general	Activado/a – No activado/a
Activación negativa	Tenso - Relajado	Estrés	Con presión – Sin presión
Ansiedad	Nervioso/a – Tranquilo/a	Ansiedad cognitiva	Preocupado/a – Tranquilo/a
		Cohesión	Desconectado/a del equipo – Integrado/a

	**TEP 2010**		**Adaptation TEP 2019**	

**Variables**	**Opposing adjectives**	**Variables**	**Opposing adjectives**

General tiredness	Tired – Fresh	Rest	Tired – Energetic
Self-confidence	Confident - Not Confident	Self-confidence	Confident - Not Confident
Motivation	Motivated - Unmotivated	Motivation	Motivated – Unmotivated
Concentration	Focused – Scattered	Concentration	Focused – Scattered
Hostility	Calm – Angry	Hostility	Calm – Angry
Weariness	Happy – Sad	Mood state	Happy – Sad
Positive arousal	Activated - Not activated	General arousal	Activated - Not activated
Negative arousal	Tense – Relaxed	Stress	Under pressure – Not under pressure
Anxiety	Nervous – At ease	Cognitive anxiety	Worried – At ease
		Team cohesion	Disconnected from team - Integrated

*First, we show the Spanish original adjectives, and after these, the English translation.*

### Participants

The procedure used to obtain the sample was “snowball” probabilistic sampling. To this end, we began by contacting professionals related to different sporting disciplines that work in institutions and/or teams. They helped us to recruit participants that fit the profile outlined from among their acquaintances and these, in turn, helped is to find other potential participants among their acquaintances. The inclusion criteria were (a) that they were involved in a team sport, (b) were over 16 years old, and (c) that their mother tongue was Spanish.

The total number of participants was 309 men and women aged between 16 and 53 years old (M: 22.5; and SD: 7.2). The sample was divided randomly into two groups. Therefore, part of the sample was used for Exploratory Factorial Analysis (sub-sample A) and the other for Confirmatory Factorial Analysis (sub-sample B). Table 2 presents the specific data associated with each sub-sample (EFA and CFA).

**TABLE 2:** Sample characteristics.

	**Sub-Sample A**	**Sub-Sample B**
**Sample size (n)**	199 participants (M age = 23.29/S.D. = 7.62)	110 participants (M age = 22.47/S.D. = 6.56)
Women	33 participants (16.59%) (M age = 25.90/S.D. = 8.95)	11 participants (10%) (M age = 23.54/S.D. = 5.53)
Men	166 participants (83.41%) (M age = 22.50/S.D. = 7.02)	99 participants (90%) (M age = 22.35/S.D. = 6.65)
Years of practice	M = 13,98/S.D. = 6.41	M = 14.68/S.D. = 6.25
Sports	Soccer = 86.93% / Basketball = 12.06% Baseball = 0.5% / Handball = 0.5%	Soccer = 94.54% /Field Hockey = 2.72% / Volleyball = 1.81% / Indoor Soccer = 0.90%
Professionals vs. Amateurs	Professionals = 30.20% Amateurs = 69, 8%	Professionals = 25.45% Amateurs = 77, 27%

### Procedure

All the participants were given the online version of the TEP. We composed a brief explanatory message which we distributed through mobile messaging applications and which included a link to the website where the TEP was hosted. Accessing this site brought participants to: a presentation and explanation of the research objectives, information related to data protection based on the Spanish Organic Law concerning the Protection of Personal Data and Guarantee of Digital Right (Boletín Oficial del Estado, 2020); and a brief questionnaire concerning demographic aspects and details of interest for the classification of the sample. Finally, in order to participate in the study, the athletes had to accept all its terms and conditions. Having done so, the participants gained access to the TEP. After completing it they received (via the email address they had given us) their individualized profile along with the results of their psychological state and a brief explanation to assist interpretation.

### Data Analysis

With regard to the main objective of this study, conducting the study of the psychometric properties of the TEP, the internal structure of the test has been studied. This was conducted via a cross validation process (Lasa et al., 2008).

With sub-sample A, an Exploratory Factorial Analysis was carried out using the Principal Component extraction method. This method was chosen in hopes of maximizing the degree of variance explained by the variables, in this way ensuring a factorial solution that is as representative as possible. The application scenarios were verified using the Kaiser-Meyer-Olkin Measure of Sampling Adequacy and the Bartlett Sphericity Test was also carried out on sub-sample A. In order to facilitate the interpretation of the significance of the selected factors, a Varimax rotation with Kaiser normalization was conducted (Kaiser, 1958). This implements an orthogonal rotation of the factorial axes based on the independence at a theoretical level as has been mentioned previously with respect to each of the factors. In addition, internal consistency was analyzed based on the Cronbach reliability index. For statistical analyses, we used the IBM SPSS Statistics 25 Software.

With sub-sample B, a Confirmatory Factorial Analysis (CFA) was carried out to estimate the parameters and evaluate the fit of the model resulting from the EFA. In this statistical test, the null hypothesis established that the proposed theoretic model is adjusted to the model resulting from the EFA data. If the null hypothesis is rejected, the proposed theoretic model is not adjusted to the model resulting from the EFA data. The Robust Maximum Likelihood method (RML) was used. The RML method can be applied when the variables observed are of a continuous nature and the data does not follow a normal distribution. In comparison to other estimation methods used in the CFA with ordinal variables, the RLM method (together with the Robust Unweighted Least Squares method, RULS) has demonstrated a better performance with fewer Type I error values (Holgado-Tello et al., 2018). We have used the LISREL 9.2 Software to carry out this analysis.

Related to the second objective, an analysis of the Student’s *T* test for independent samples with an abnormality correction (Z) was carried out to evaluate the existence of significant statistical differences in relation to the level of dedication variable. Additionally, a MANOVA was carried out to find out if significant differences between the TEP psychological variables, depending on the level of dedication and the gender of the participants as well, as the interaction between both variables exist, considering each of the 9 TEP variables, as dependent variables; and the level of dedication (amateurs and professionals) and the gender (women and men) as independent variables.

## Results

### Sample Normality Analysis and Kurtosis

The Kolmogorov-Smirnov Normality test indicates that the sample does not follow a normal distribution (*p* = 0.000 and d.f. = 200, in all items), so we opted to use non-parametric tests. In relation to Kurtosis or Asymmetry, all items obtained values which were considered adequate (<1.5 and -1.5), except for the item referring to Unity, which presented greater asymmetry (2.28). See Table 3.

**TABLE 3:** Results of normality test and Kurtosis.

	**Kolmogorov-Smirnov**			**Kurtosis**	**Standard error**
	**Statistics**	**gl**	**Sig.**		
Self-confidence	0.153	200	0.000	0.79	0.34
Motivation	0.163	200	0.000	0.68	0.34
Team cohesion	0.232	200	0.000	2.28	0.34
Concentration	0.133	200	0.000	0.12	0.34
Mood state	0.169	200	0.000	0.26	0.34
Rest	0.173	200	0.000	−0.73	0.34
General arousal	0.092	200	0.000	−0.99	0.34
Cognitive anxiety	0.099	200	0.000	−0.55	0.34
Hostility	0.125	200	0.000	0.48	0.34
Stress	0.117	200	0.000	−1.05	0.34

### Internal Consistency Analysis

Lastly, internal consistency was analyzed using Cronbach’s Alpha Coefficient. The result showed a value of α = 0.823, taking into account all the variables of the TEP. As regards the individual factors, Factor 1 showed an internal consistency of α = 0.851 and Factor 2 of α = 0.726.

### Exploratory Factor Analysis (EFA)

In the initial EFA, it was found that the Hostility variable shared part of its variance with the two factors proposed by the EFA (Factor 1 = 0.60 and Factor 2 = 0.36), which indicated that its factorial structure was not clear. It was therefore decided that the Hostility variable be removed. It was also found that the results of the weights of the other variables as regards the factors do not present significant changes and the percentage of variance explained increased from 59.5% to 61.4% (see Table 4).

**TABLE 4:** Total variance explained by the TEP items.

**Comp.**	**Initial self-values**			**Sums of the squared saturations of the extraction**			**Sums of Squared saturations of rotation**		
	**Total**	**% de Variance**	**% Accumulated**	**Total**	**% de Variance**	**% Accumulated**	**Total**	**% de Variance**	**% Accumulated**
1	3.773	41.921	41.921	3.773	41.921	41.921	3.553	39.479	39.479
2	1.753	19.475	61396	1.753	19.475	61.396	1.972	21.916	61.396
3	791	8.792	70.188						
4	627	6.968	77.156						
5	524	5.819	82.975						
6	497	5.523	88.497						
7	404	4.487	92.985						
8	361	4.008	96.993						
9	271	3.007	100.00						

**FIGURE 1:**
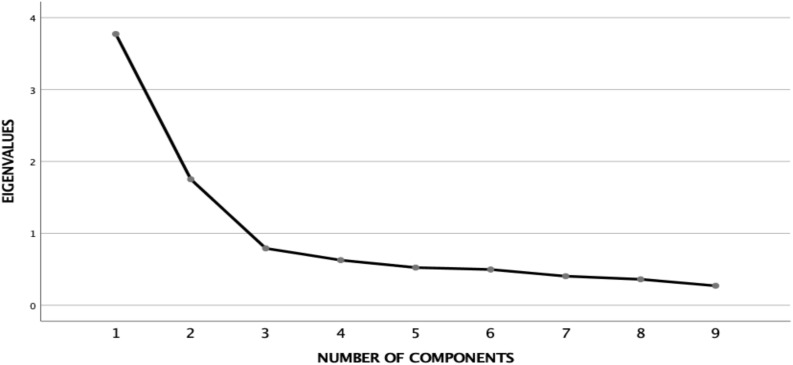
Sedimentation graph.

Under these conditions, the result of the KMO (Kaiser-Meyer-Olkin) index was 0.843, suggesting that the data were considerably interrelated (≥0.84). Meanwhile, the results of the Bartlett Sphericity Test confirmed the applicability of the Factorial Analysis (Chi-square = 753.933; d.f. = 45, and *p* = 0.000). Two factors were obtained which explained 61.3% of the variance. Table 5 shows the matrix of rotated components in which the clustering of the variables around the two factors can be observed. The sedimentation graphic can also be seen in Figure 1.

**TABLE 5:** Matrix of rotated components and communality.

**Variables**	**Components**		**h2**
	**1**	**2**	
Motivation	**0.827 (F1)**	0.020	0.685
Self-confidence	**0.804 (F1)**	0.117	0.660
Mood state	**0.777 (F1)**	0.210	0.648
Concentration	**0.726 (F1)**	−0.42	0.529
Team cohesion	**0.692 (F1)**	0.094	0.488
Rest	**0.695 (F1)**	0.086	0.490
General arousal	0.011	**0.835 (F2)**	0.697
Cognitive anxiety	0.359	**0.759 (F2)**	0.705
Stress	−0.025	**0.789 (F2)**	0.624

*The bold type indicates the higher values in each factor. h2 = Communalities.*

The results demonstrated a factorial structure consisting of two principal factors and reliability coefficients, both globally and at the factor level. The items were distributed around these factors, giving rise to one factor which includes variables which have a positive relationship with performance (Self-confidence, Motivation, Concentration, Rest, Team cohesion, and Mood state), and another with variables which negatively affect performance (General arousal, Stress, and Cognitive Anxiety). The relationship, positive or negative, between each variable and performance is in line with the findings of other studies in assessing this effect in athletes (as detailed in the Discussion section).

### Confirmatory Factor Analysis (CFA)

**FIGURE 2:**
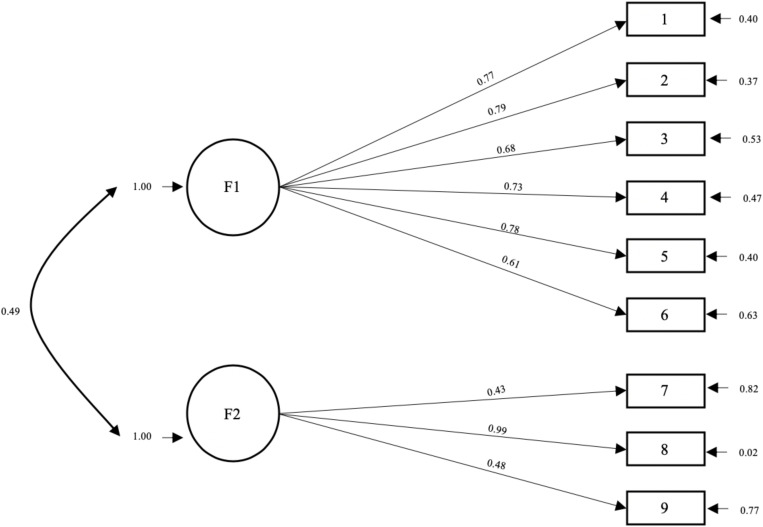
Overview of the confirmatory factorial analysis results.

According to the results obtained through the EFA, a first-rate factorial model, consisting of two factors, is proposed. The values obtained through the confirmatory factor analysis on the second sub-sample (B) indicated an appropriate model fit. Thus, with a confidence level of 99% the proposed model will be accepted according to the chi-squared test. The main global indices of fit goodness are: χ^2^ Satorra-Bentler (d.f. = 26; p = 0.0232) = 37.99; RMSEA = 0.08 with a confidence interval of 90% between 0.032 and 0.122; RMR = 0.085 and GFI = 0.91. The following incremental indices were also analyzed: CFI = 0.95 and NNFI = 0.93. The model was specified as shown in Figure 2.

The results obtained confirm the two-factor structure proposed by the EFA. Thus, we could deduce that it is possible to get a TEP profile which would define the psychological state which makes it easier for athletes to use all their resources to deal with the sporting situation they face. This profile would involve high levels of factor 1 variable (performance enhancing variables; motivation, mood, rest, concentration, self-confidence and team cohesion); together with low levels of factor 2 variables (performance limiting variables: cognitive anxiety, stress and general arousal). These results would be in line with those described by Mouloud and El-Kadder (2016), who after conducting a bibliographic review in relation to the psychological characteristics of elite athletes, found that performance was enhanced when athletes present high levels of self-efficacy (self-confidence) and motivation, and low levels of cognitive anxiety.

### Comparison of Profiles of Amateur and Professional Athletes

We used the Student’s *T* test to carry out this analysis on the independent samples. We initially standardized the scale of the data to Z-scores to correct the sample’s abnormality, and from these scores, we carried out the Student’s *T* test and found the size of the effect by analyzing Cohen’s d. The results indicated significant differences between both groups (amateurs vs. professionals) in the motivation variable (*p* = 0.05 and d = −0.2). The group of professional athletes obtained higher scores, which means that professional athletes presented higher levels of motivation than amateur athletes in the moments leading up to competition. The results show small size effect (≤0.2) in motivation following the Cohen’s guidelines for *d* (Cohen, 1988). Data obtained from this analysis of each of the TEP variables is presented in Table 6.

**TABLE 6:** Summary of data comparison by group, Amateurs vs. Professionals.

		**Levene’s Test for Equality of Variances**		**t-test for Equality of Means**						
		**F**	**Sig.**	**t**	**df**	***Sig. (2-tailed)* p**	***Mean Difference (d Cohen)***	**Std. Error Difference**	**95% Confidence Interval of the Difference**	
									**Lower**	**Upper**
SELF-CONFIDENCE (Z score)	Equal variances assumed	0.92	0.33	−1.02	307	0.30	−0.13	0.13	−0.38	0.12
	Equal variances not assumed			−0.97	145.03	0.33	−0.13	0.13	−0.39	0.13
MOTIVATION (Z score)	Equal variances assumed	0.82	0.36	−1.89	307	**0.05**	−0.24	0.12	−0.48	0.01
	Equal variances not assumed			−1.95	172.57	**0.05**	−0.24	0.12	−0.47	0.00
TEAM COHESION(Z score)	Equal variances assumed	1.07	0.30	−0.49	307	0.62	−0.06	0.13	−0.31	0.18
	Equal variances not assumed			−0.53	187.89	0.59	−0.06	0.12	−0.29	0.17
CONCENTRATION (Z Score)	Equal variances assumed	0.89	0.34	−0.86	307	0.38	−0.11	0.13	−0.36	0.14
	Equal variances not assumed			−.83	148.24	0.40	−0.11	0.13	−0.37	0.15
MOOD STATE (Z Score)	Equal variances assumed	1.59	0.20	1.14	307	0.25	0.14	0.12	−0.10	0.39
	Equal variances not assumed			1.08	143.93	0.27	0.14	0.13	−0.12	0.40
REST (Z Score)	Equal variances assumed	1.82	0.17	0.90	307	0.36	0.11	0.13	−0.13	0.36
	Equal variances not assumed			0.86	147.44	0.38	0.11	0.13	−0.15	0.37
GENERAL AROUSAL (Z Score)	Equal variances assumed	0.00	0.99	−1.00	307	0.31	−0.13	0.13	−0.37	0.12
	Equal variances not assumed			−0.99	155.95	0.32	−0.13	0.13	−0.38	0.12
COGNITIVE ANXIETY (Z Score)	Equal variances assumed	0.33	0.56	−0.00	307	0.99		0.13	−0.25	0.25
	Equal variances not assumed			−0.00	153.04	0.99	−0.00	0.13	−0.25	0.25
STRESS (Z Score)	Equal variances assumed	0.05	0.81	−0.47	307	0.63	−0.06	0.13	−0.31	0.19
	Equal variances not assumed			−0.47	156.59	0.63	−0.06	0.13	−0.31	0.19

In the MANOVA test, the multivariate contrasts obtained using Pillai’s Trace indicate that there is no difference in the interaction of dependent variables in relation to the interaction between gender + the level of dedication (F[9,297] = 1.82, *p* = 0.066), nor in relation to the gender variable alone (F[9,297] = 1.08, *p* = 0.375) nor to the level of dedication (F[9,297] = 1.85, *p* = 0.059). However, differences in the dependent variables were found separately: in the self-confidence variable in relation to gender (F[1,305] = 3.95, *p* = 0.048), in the motivation variable in relation to the level of dedication (F[1,305] = 9.57, *p* = 0.002); and in the motivation (F[1,305] = 5.93, *p* = 0.015) and rest variables (F[1,305] = 7.06, *p* = 0.008) in the interaction between gender and level of dedication. In general, men present higher levels of self-confidence than women, with a difference of 6.75 points on the VAS scale (95%CI = -13.43, -0.069); for the level of dedication the levels of motivation are higher in professional athletes, with a difference of 11.63 points on the VAS scale (95%CI = -19.02, -4.23); and the analysis of interaction between both variables found that the level of motivation was higher in professional female athletes, 20.79 points on the VAS scale (95%IC = 7.72, 33.85; *p* = 0.002); and the level of rest in amateur male athletes was higher, 7.44 points on the VAS scale (95%IC = 0.25, 14.62; *p* = 0.043). No other differences were found.

## Discussion

This paper aims to check the characteristics of the new version of the Psychological State Test (TEP) developed by Buceta (2010) for team sports in Spanish. The main objective of the TEP is to evaluate the “pre-competitive psychological state” construct, for which the psychological variables that have been most studied and that have the greatest consensus in terms of their relationship and influence on sports performance were taken into account. This new version of the TEP was a response to the problems encountered by athletes in interpreting some of the adjectives used in the initial TEP. Accordingly, a pilot study was carried out with the collaboration of sports psychology experts in which several adjectives were modified so as to be more in line with the athletes’ usual vocabulary. The reconceptualization of three of the scales from the original TEP was also carried out. These were General arousal, Stress and Cognitive Anxiety.

Regarding internal consistency, the TEP was found to be reliable (α > 0.80). As a general criterion, George and Mallery (2003) consider a coefficient α greater than.80 as “good”. In regard to each individual factor, Factor 1 would also have a good internal consistency (>0.80) and Factor 2 would have an acceptable internal consistency (in the classification established by these authors) of >0.70. The result of the Exploratory Factorial Analysis specified a structure of two main factors. Factor 1 is composed of six scales with weights between 0.69 and 0.83 and Factor 2 is composed of three scales, with weights between 0.76 and 0.83. This factorial solution explains 61.4% of the variance explained. This two-factor model was supported by results obtained through a Confirmatory Factorial Analysis.

Factor 1 would consist of the scales: Self-confidence, Motivation, Concentration, Rest, Team cohesion, and Mood state. These scales could be considered within the performance facilitators. Based on the related scientific literature the high scores on these scales could be interpreted as positive regarding psychological state profile prior to competition in sports teams.

Some examples are as follows: athletes with high levels of self-confidence/self-efficacy tend to be involved for the greatest length of time, to have a higher level of effort and to persist in order to achieve their goals (Dosil, 2008). Meanwhile, motivation is considered essential in order for athletes to acquire the commitment, perseverance and tolerance of frustration which competition demands. Therefore, it is considered that it should be high in athletes in order to assist performance (Buceta, 2003). High levels of concentration facilitate the implementation of strategies and resources for dealing with competitive events. According to Dosil (2008) concentration is key for athletes attaining their optimum performance as well as for facilitated learning. In relation to the level of perceived Rest, high levels in this variable can stimulate the athletes’ participation in the sporting activity. Studies conducted with the POMS determine that when athletes presented high scores on the Fatigue scale, this was related to a reduction in physical capacity and the athletes’ perception of personal effectiveness (Terry, 1997). Hanin (2000b), meanwhile, found that soccer players considered feeling motivated, confident and alert (attention) as states which assisted performance. In the same study, the players identified feeling tired and insecure as states which impaired their performance. Lastly, in relation to the variable of Team cohesion, Carron et al. (2002) carried out a meta-analysis of 46 studies which looked at the association between unity and sporting success. The results confirmed the positive relationship (from “moderate” to “significant”) between these variables. The more cohesive teams tended to have more success and the successful teams were more likely to develop a sense of unity.

On the other hand, “Factor 2” would be composed of the variables General arousal, Cognitive Anxiety, and Stress. In this case, we could consider that high scores on these scales can restrict the implementation of the athletes’ resources and skills and therefore adversely affect their performance based on the related scientific literature.

Some examples of these studies are as follows: in relation to General arousal, the results obtained in our study are in line with the inverted “U” theory of Yerkes and Dodson (1908). These authors postulate that a higher level of performance could be attributed to medium levels of arousal, with the highest and lowest levels assisting performance the least. On the other hand, Cognitive anxiety (which refers to the degree to which a person worries or has negative thoughts) and Stress are considered as non-functional in relation to sporting performance (Weinberg and Gould, 2007; McGrath, 1970). In the Multidimensional Anxiety Theory, the authors Martens et al. (1990b) argued that anxiety may have an impact on attention, concentration and athletes’ decision making. In terms of correlation, the authors stated that cognitive anxiety has a negative linear relationship with performance. In other words, the higher the levels of cognitive anxiety, the worse the performance. The ideal profile to benefit performance in this regard would involve athletes presenting medium levels of General arousal and low levels of Cognitive Anxiety and Stress.

We can conclude the existence of this optimal psychological profile that appears to facilitate sports performance, defined by high levels of Factor 1 variables and moderate and low levels of the variables grouped around factor 2. This follows the findings of Larumbe (2006) in his studies on the PODIUM in which he describes a “positive psychological disposition” among marathon runners characterized by high levels of self-confidence and motivation and with controlled arousal levels and anxiety.

The Hostility scale was not clearly associated with any of the resulting factors. Lane and Terry (2005) explained why the factors of stress and hostility are associated with good performance in some studies and not in others. According to these authors, depressive mood determines the functional impact of stress and hostility on performance. Without the presence of depressive symptoms, pressure and hostility contribute to increasing the athletes’ determination. However, with depressive symptoms, stress and hostility did not benefit performance. We can conclude that hostility can mobilize athletes and lead to greater perseverance and willingness to compete with all their available resources. On the other hand, high levels of hostility may be related to greater difficulty controlling general arousal and therefore to issues regarding maintaining focus and being precise with their movements or technical actions, and/or lead to greater impulsivity in making decisions. The apparent need (based on the result of the factor analysis) to assess the effect of individual hostility on each athlete, leads us to conclude that it is not a good scale to use to assess the collective disposition of teams, and so it was removed from the instrument.

The second objective of this study was to look at whether there were significant differences as regards the psychological profile of amateur athletes and professional athletes. In this regard, we can conclude that professional athletes presented higher levels of motivation. In line with our findings, Modroño and Guillén (2006) did find significant differences in motivation levels between competitors and non-competitors, with the levels of extrinsic motivation found to be higher in competitors. It is important to understand that it is in the competition where windsurfers can win cash or material prizes, which may explain this increased level of extrinsic motivation; hence, it can be compared to the prizes or remuneration awarded to professionals in team sports, as is the case with our sample. One would expect, in such cases, that motivation levels would therefore be higher than those of amateur athletes. In this sense, Carpenter and Yates (1997) carried out a study on amateur and semi-professional football players, the authors found that semi-professional football players, when compared to amateurs, considered the financial and status enhancements of their sport to be the main reason for playing. Halldorsson et al. (2012), reported that elite athletes report higher levels of motivation and commitment than non-elites. Mallett and Hanrahan (2004) found that Olympic and World Championship level athletes exhibit self-determined forms of motivation, and are achievement oriented, highly driven, and self-believing. If we consider the gender variable, our results found that men presented higher levels of self-confidence than women, in line with the findings of a study on recreational runners carried out by Larumbe-Zabala et al. (2019).

With regard to the possibilities of practical applications of the TEP, several studies related to the advice to coaches and technical bodies in the design and management of the pre-match talks are worth highlighting. Vargas-Tonsing and Bartholomew (2006) studied the effect that pre-match talks had on the athletes’ perception of self-efficacy. Their findings could not confirm that the various pre-match talks analyzed resulted in any significant effect on the participants’ levels of self-efficacy. They concluded that in order for pre-match talks to have a positive effect on performance (to improve the players’ levels of self-efficacy), the coaches had to be aware of the emotional intensity of the athletes prior to the talk in order to avoid generating states of over-arousal or anxiety. They concluded that it was very important for the coach to be aware of the players’ prior emotional state in order to thus tailor their talk and achieve beneficial effects which stimulate the appropriate arousal levels.

In this regard, the TEP can be a useful tool for advising coaches on the psychological state of their teams prior to competition. One practical application in this regard was that presented by Díaz-Tendero et al. (2018) involving a field hockey team. The results showed that the percentage of times the coach used the information provided by the TEP in his pre-game talk was 92%. The evaluation obtained by the technical team after each game regarding the usefulness of this information was an average of 7.8 points (on a scale of 0 to 10), and the degree to which, according to the players, the “team profile” matched their perception of the team was an average of 8.1 points (on a scale of 0 to 10). In this case, the method used to complete the TEP was text messaging (SMS) via mobile phones. However, this system had many practical limitations with regard to completing the questionnaire and the delay in receiving the results from the players and the coach/coaching staff. To address these limitations, we used the online version of the TEP in this study.

In recent years, the number of apps and online resources which support psychological intervention tools has grown exponentially. Concepts such as cognitive ergonomics (which encompasses the psychological aspects of people’s interaction with technology) and usability (a discipline which studies the processes involved in people’s interaction with interactive products in order to facilitate their use), are key when it comes to designing and evaluating technological tools to assist interventions or evaluations in the applied field of sports psychology. In future research projects, these aspects should be looked at more specifically as regards the development of an application for smartphones, and other mobile devices, which would support the TEP. This app could make it easier for athletes to receive notifications in order to complete the questionnaire and to store the results of these measurements of the psychological state of each player. It could also provide coaching staff with an access profile: in this section, the psychological profiles of the team would be stored for analysis and for the potential integration of this information, along with that from other areas related to sporting performance.

The TEP has proved to be a reliable tool for assessing pre-competitive psychological states in team sports. Unlike other tools that attempt to evaluate the same construct, the TEP provides information on a larger number of variables by broadening the profile that can be obtained from athletes compared to, for example, the CSAI-2R (Andrade et al., 2007), which only provides information on three variables (somatic anxiety, cognitive anxiety and self-confidence). Another advantage that the TEP presents over other tools is the minimal time required for completion, which facilitates the precision of self-observation that athletes need to respond to this type of test in those pre-competitive times when higher levels of anxiety (both somatic and cognitive) are detected (Martens et al., 1990b). Furthermore, an innovative contribution to the online version of TEP used for this study is the automation of the immediate correction and feedback that athletes receive. This is a great advantage in that it allows sports psychologists to work with the athletes on their mental preparation prior to a competition, as well as facilitating self-regulation by the athletes themselves.

However, this study presents a number of limitations that should be taken into account. The sampling was done using the snowball method and not a simple random sampling which could give more solidity to the data. On the other hand, the sample used in the two factorial analyses (EFA and CFA) was collected at the same time, but it is more appropriate to collect the data consecutively. To support the conclusions regarding the differences between amateurs and professionals, it would be advisable to expand the sample of professionals in order to have matching numbers of participants from both groups.

For future research projects, we think it is important to evaluate the predictive validity of the TEP so that it can be a useful tool when predicting behaviors related to athletes’ performance. In this line, another aspect to conduct further research on is the usefulness of coaching programs for the psychological management of teams based on the psychological state profiles provided by TEP.

## Data Availability Statement

The raw data supporting the conclusions of this article will be made available by the authors, without undue reservation.

## Ethics Statement

The studies involving human participants were reviewed and approved by Commission of the PhD program in Health Psychology and Bioethics Commission of National University of Distance Education (UNED). Written informed consent to participate in this study was provided by the participants’ legal guardian/next of kin.

## Author Contributions

PD-T, MP-L, and AL performed conceptualization, methodology, and investigation. AL performed formal analysis and wrote, reviewed, and edited the manuscript. PD-T and MP-L performed resources and data curation. PD-T wrote original draft. MP-L performed supervision. All authors contributed to the article and approved the submitted version.

## Conflict of Interest

The authors declare that the research was conducted in the absence of any commercial or financial relationships that could be construed as a potential conflict of interest. The handling Editor declared a shared affiliation with the authors at time of review.
